# Chemical Composition and Safety of Unrecorded Grain Alcohol (Bai Jiu) Samples from Three Provinces in China

**DOI:** 10.3390/ijerph15122710

**Published:** 2018-12-01

**Authors:** Ian M. Newman, Ling Qian, Niran Tamrakar, Bo-Bo Zhang

**Affiliations:** 1 Nebraska Prevention Center for Alcohol and Drug Abuse, Department of Educational Psychology, University of Nebraska-Lincoln, P.O. Box 880345, Lincoln, NE 68588, USA; ntamrakar1987@gmail.com; 2 National Center for Health Education, Beijing 100011, China; qianlingzh@126.com; 3 School of Biotechnology, Jiangnan University, Wuxi 214122, China; bobozhang@jiangnan.edu.cn

**Keywords:** noncommercial alcohol, Chinese liquor, 白酒, white spirits, artisanal liquor, contamination, adulteration, unfair trade

## Abstract

About 20% of spirits consumed in China are “unrecorded”, where these spirits are produced in small-scale distilleries and sold outside the systems of taxation and quality control. Researchers visited small distilleries in rural Yunnan, Hubei and Anhui and purchased 56 samples of unrecorded bai jiu. Seven samples of the recorded bai jiu were purchased as reference samples. An independent laboratory conducted a blind analysis of the samples. Results were compared to the standards for unrecorded alcohol adopted by the European Commission’s Alcohol Measures for Public Health Research Alliance (AMPHORA). No samples exceeded the AMPHORA guidelines for methanol, ethyl acetate, lead and cadmium; one sample exceeded 1000 g/hL of combined higher alcohols; one sample exceeded 100 mg/L of arsenic; and three samples exceeded 50g/hL of acetaldehyde, but only by relatively small amounts. Low-priced unrecorded bai jiu averaged 9.8 RMB/jin (500 mL), compared to 10.7 RMB/jin for inexpensive recorded bai jiu. The low-priced unrecorded bai jiu samples had a mean alcohol-by-volume of 51.8%, compared to 50.1% for the recorded bai jiu samples. The results did not raise any critical safety issues with unrecorded bai jiu, but there may be long-term health risks related to ethanol, acetaldehyde and arsenic. The social ties between the bai jiu makers and the people who consume their product are a deterrent to adulteration; but when bai jiu is sold outside of the social circle, the deterrent disappears.

## 1. Introduction

This paper describes the chemical analysis of 56 samples of unrecorded bai jiu (distilled liquor) produced in small unregistered production sites in central China and seven samples of recorded bai jiu purchased from grocery stores. Unrecorded bai jiu is produced outside of regulation, testing and/or taxation. Both the public health community and the recorded alcohol industry are advocating for restrictions on the production of unrecorded bai jiu based on their concerns about possible contamination or adulteration and because untaxed bai jiu is often cheaper than recorded alcohol. The production of distilled bai jiu is a traditional occupation in China. Making, buying and consuming unrecorded alcohol is not illegal. Because production is not taxed or officially reported it is difficult to estimate the per capita consumption of this type of bai jiu. The World Health Organization estimates that 67% of recorded alcohol in China is consumed in the form of spirits (bai jiu), and over 20% of the spirits consumed in China are made in unregulated distilleries (unrecorded alcohol) [1] (p. 316).

### 1.1. Risks and Benefits of Unrecorded Alcohol Worldwide

The term unrecorded alcohol is a broad category that can include legal or illegal homebrews made in unregistered factories, alcohol that is legally or illegally imported without a record, alcohol products not intended for human consumption but nevertheless consumed by humans (surrogate alcohol), and unrecorded alcohol that is added to branded products for the purpose of counterfeiting higher-cost brands [1] (pp. 112–114) [2].

The absence of government regulation and inspection of unrecorded alcohol beverages creates concern among public health authorities and opposition from the recorded alcohol industry [1] (p. 112) [3]. The public health concerns are two-fold: One concern is about intentional or unintentional contamination. In its most egregious form, the intentional adulteration of alcohol beverages to enhance their potency or appeal can lead to poisoning or death. On the other hand, unintentional errors in distillation or accidental contamination by elements in the environment may create a lifetime risk of illness in users who consume the product over the long term. Because unrecorded alcohols are not regularly tested for safety, this type of contamination would not be detected or corrected. The second public health concern is that the low price and high availability of alcohol contributes to the over-use of alcohol, which burdens societies with social, legal, economic and health problems. A systematic review of unrecorded alcohol studies [4] concluded that unrecorded alcohol generally was cheaper than branded alcohol, which exacerbated alcohol problems, especially in low-income communities [4]. Because unrecorded alcohols are produced and sold informally, it is difficult to restrict access by place, time of sale or age of customer. When added to branded products, as in counterfeiting, unrecorded alcohols threaten the reputation of recorded, branded alcohol. Unrecorded alcohol in many places is not taxed; consequently, in addition to a loss of revenues to governments, there is an unfair price advantage for unrecorded alcohol compared to recorded alcohol. In some countries, the potential to profit from illegal alcohol production has led to increases in corruption and crime [5]. Both the alcohol industry and the public health sector have called for the elimination of unrecorded alcohol [6] or bringing it into the taxation and monitoring systems [1] (p. 112).

It is difficult to separate the risks of unrecorded alcohol from those of recorded alcohol, since many drinkers drink both types. Studies of unrecorded alcohol found that, with the possible exception of the tax and crime concerns, the burden on societies from unrecorded alcohol was the same as presented by recorded alcohol [2,4].

On the beneficial side of the ledger, manufacturing unrecorded alcohol is a traditional occupation in many places in the world; it adds value to agricultural products, often provides income for women and the spent grains are used to feed livestock. To summarily end informal alcohol manufacturing would affect the economic wellbeing of a significant number of people. The World Health Organization’s Global Strategy to Reduce Harmful Use of Alcohol [7] recognizes the economic value provided by small-scale distilleries. In countries where the production, sale and use of unrecorded alcohol is legal, the citizens are spared the heavy costs that would result from an attempt to ban it. The health benefits of light alcohol use by healthy individuals are currently being debated, with some studies declaring even light use of alcohol to be problematic for the majority of people [8], and other studies producing some evidence of health benefits from light use of alcohol in healthy individuals (at least for men, e.g., Reference [9]). In China, light alcohol consumption has for centuries been regarded as beneficial for the prevention and treatment of many health conditions, and clinical reports provide some evidence of beneficial effects of light consumption [10].

### 1.2. Production

Bai jiu production in small village factories in China follows traditional practices that have been used for centuries [10,11,12]. Chinese bai jiu is made most frequently from sorghum or rice. Grain is heated with steam until the husk cracks. After it has cooled to the appropriate temperature, yeast is added. The grain is held at a constant temperature for about a day. After a day, the fermenting grain is transferred to large holding containers for 10 to 15 days to complete fermentation. The fermented grain is then heated with steam and the evaporate is distilled. This method, referred to as the dry method, was used at all but two of the sample sites in this study. An alternative method mixes the grain and yeast in water before holding for 10–15 days for fermentation, and then it is reheated and the evaporate is distilled. Distillation involves directing the steam through a length of pipe that is cooled by water and collecting the condensate. Because alcohols have lower evaporation points than water, the first fraction of the condensate contains a higher concentration of ethanol, other alcohols and acetaldehyde compared to the later fractions of the condensate. Some makers separate the first fraction and discard it. Other makers mix some of the first fraction back into the spirits to increase the alcohol-by-volume (ABV). The type of grain, the composition of the yeast, the materials and quality of the equipment (containers, stills, pipes, etc.) and the skill of the maker all have an impact on the quality of the product. The used grain is often used for animal food. Some of the used grain may be retained and mixed with the next batch of bai jiu. Following distillation, bai jiu is aged in large ceramic containers with airtight seals, often buried in the ground to stabilize the temperature. Bai jiu is typically sold directly from the manufacturing site or through small local shops and restaurants. The manufacturing site is often also the residence of the makers’ family. Occasionally makers will sell their product to a larger company that collects from many makers, then blends the products together before further refinement and sale. The village-level production of unrecorded bai jiu in China has been described by Qian et al. [12] and in Vietnam by Luu, Nguyen and Newman [13].

### 1.3. Cultural Significance

The word bai jiu is the romanization of 白酒. Bai meaning transparent, and jiu means alcohol. Bai jiu is sometimes referred to as a white spirit. Bai jiu’s historical origin is unclear. Some say bai jiu originated in the Yuan Dynasty [10,14], but the first clear reference to a distilled spirit made from wheat, barley or rice is found in the text Song Shi written around 928 CE [10,15]. While bai jiu’s historical origin may be uncertain, its importance in Chinese culture is not. Bai jiu is a beverage that is also considered a food and typically accompanies meals at home, especially in rural areas. Its presence is also important at festival banquets, rites of passage and other special celebrations. Bai jiu given as a gift signifies wishes for happiness and good health. Special national brands of bai jiu such as Mao Tai and Wu Liang Ye are symbols of national pride and are important in diplomacy. Perhaps the most memorable diplomatic occasion facilitated by Mao Tai bai jiu was the banquet toasting of Richard Nixon by Zhou Enlai in February 1972.

### 1.4. Safety of Unrecorded Bai Jiu

Currently the most widely-used guideline for the maximum safe levels of compounds in unrecorded beverage alcohol comes from the European Commission’s Alcohol Measures for Public Health Research Alliance (AMPHORA) [16]. Use of the AMPHORA guidelines allows for comparisons of unrecorded alcohols from different countries. In their systematic review on worldwide consumption and the toxicology of unrecorded alcohol, Rehm et al. [4] acknowledged the scarcity of information about unrecorded alcohols in China and considered this an area needing exploration. Their systematic review of chemical analyses of unrecorded alcohol [4] did not include any alcohol samples from China.

This paper reports results of the chemical analysis samples of unrecorded bai jiu produced in small, family-operated factories in rural areas of Yunnan, Anhui, and Hubei and seven samples of recorded bai jiu were purchased in convenience stores in Beijing. The recorded bai jiu samples served as a reference group.

Only two previous studies have examined the chemical composition of Chinese unrecorded bai jiu [17,18]. 47 samples collected from around Xianning in the Hubei province [17] had ethanol concentrations ranging from 38.7–63.7%, with 97.9% of the samples possessing a >40% ABV. The sum of six higher alcohols ranged from 4.7–201.1 g/hL pa. None of the samples contained methanol and methyl acetate levels exceeding the AMPHORA guidelines. Three samples contained acetaldehyde at levels higher than the AMPHORA guidelines. One sample exceeded the guideline for lead, and two samples exceeded the guideline for cadmium. The second study [18] reported on the analysis of 61 samples from rural villages throughout central China. The ABV of 95% of the samples exceeded 40% (range: 35.7–61.4%). One sample exceeded the guidelines for higher alcohols. Concentrations of methanol and ethyl acetate, lead, arsenic and cadmium were all below the AMPHORA limits. Acetaldehyde levels in 40 of the 61 samples exceeded the AMPHORA guidelines, prompting the authors to speculate this could be due to the consequences of unskilled makers failing to discard the first distillate from the distillation process. Acetaldehyde is of special significance because humans who have an inactive gene for the enzyme that metabolizes acetaldehyde are at greater risk for certain cancers when they consume alcohol regularly [19]. In China, the proportion of the population who have inherited the inactive gene may be as high as 50% [20].

## 2. Materials and Methods

### 2.1. Sample Collection

For this study, 56 samples of unrecorded bai jiu were purchased at small production sites in rural areas of Yunnan (9 samples), Hubei (24 samples) and Anhui (15 samples). Six samples of unrecorded bai jiu were purchased at two small shops in the city of Chongqing. Two samples of unrecorded bai jiu were purchased at a meat/vegetable/fruit market in Beijing. Seven samples of low-cost recorded bai jiu were purchased at convenience stores in Beijing in order to compare with the unrecorded bai jiu. Researchers purposely collected a sample of the highest and the lowest ABV bai jiu available from each vendor. All of the unrecorded alcohol samples were purchased in 1 jin (500 mL) quantities and collected in 500 mL chemically stable plastic bottles with tight lids. The recorded alcohol samples were transferred from the glass bottles to the plastic sample bottles. The sample containers were coded with a number. All samples were shipped to the laboratory for analysis within one week of purchase. Storage prior to analysis was under refrigeration.

### 2.2. Chemical Analysis

The laboratory received no information about the source of the samples. All samples were analyzed for concentrations of ethanol, methanol, acetaldehyde, higher alcohols, ethyl acetate and heavy metals (cadmium, arsenic and lead). The concentration of volatile compounds (methanol, acetaldehyde, higher alcohol, ethyl acetate) was determined by gas chromatography with a flame-ionization detector with nitrogen as the carrier gas. Inductively coupled plasma optical emission spectrometry was used to determine the concentration of heavy metals. The concentration of ethanol, reported as alcohol by volume percentage (ABV%), was determined using an alcoholometer. The details of the instrumentation and chemical analysis are reported in Appendix A. The study used the AMPHORA standard as the reference to assess the chemical safety of unrecorded alcohol [16].

## 3. Results

### 3.1. Grain, Age, Price and ABV of Unrecorded Bai Jiu Samples

The alcohol samples were made from: Sorghum (33.3% of the samples), rice (25.4%), corn (6.3%), buckwheat (4.8%) and a mixture of grains (sorghum, rice, corn, buckwheat and millet—11.1%). The source grain was not identified for 19.0% of the samples. One manufacturer used sticky (glutinous) rice. One sample was eight years old (#110), one was 5 years old (#149) and two samples were between 3 and 5 years old (#140, #143). The remainder were manufactured in the last year.

Prices ranged from 3.5–60 RMB (0.50–8.76 USD) per jin, except for the 8-year-old sample, which was 280 RMB (40.90 USD). Prices for inexpensive unrecorded bai jiu (<20 RMB/jin; <2.90 USD) ranged from 3.5–16 RMB (0.50–2.30 USD) (*n* = 34, mean = 9.8 RMB, 1.40 USD). For inexpensive recorded bai jiu, prices ranged from 6–17 RMB (0.87–2.50 USD) (*n* = 7, mean = 10.7 RMB, 1.60 USD).

The mean ABV of the unrecorded bai jiu samples was 51.8%. For the 7 recorded bai jiu samples, the mean ABV was 50.1%.

### 3.2. Chemical Composition of Unrecorded Bai Jiu Samples

A detailed table of the chemical test results is provided in Table S1 (Supplementary Materials).

#### 3.2.1. Ethanol

The ABV% for the unrecorded bai jiu samples ranged from 40.8–72.1 (40–49 ABV, 31.7%; 50–59 ABV, 60.3%; 60–69 ABV, 6.4%; 70+ ABV, 1.6%). In comparison, the 7 recorded alcohol samples ranged in ABV from 42.3–57.5%.

#### 3.2.2. How Laboratory Ethanol Concentrations Compared to Seller’s Information

When the samples were collected, we made a note of the seller’s estimate of the ABV of their product; then we compared the seller’s estimate to the ABV result from the laboratory analysis. We recorded the seller’s estimate of ABV for 56 (88.8%) of the 63 bai jiu samples. We found that 57.1% (32) of sellers correctly gave the ABV within ±2% of the laboratory test result. Fourteen (25.02%) of sellers over-stated the ABV compared to the laboratory test result, and ten (17.9%) of sellers under-stated the ABV compared to the laboratory test result. For the seven recorded bai jiu samples purchased in Beijing, we found two of these samples had higher laboratory-tested ABV levels (>2%) than reported on the bottle label.

#### 3.2.3. Methanol

Methanol was detected in all samples and ranged from 0.1 to 7.2 g/hL pa, with none of the samples exceeding the AMPHORA standard of 1000 g/hL pa [16].

#### 3.2.4. Acetaldehyde

Acetaldehyde was present in all samples, with three exceeding the AMPHORA upper limit of 50 g/hL pa [16] (Sample #104, #116, #118). The three samples exceeding the upper limit did so by relatively small amounts (66.6, 53.7, and 60.0 g/hL pa, respectively).

#### 3.2.5. Ethyl Acetate

Ethyl acetate was detected in all samples with concentrations ranging from 1.0 g/hL pa to 366.5 g/hL pa, with no samples exceeding the AMPHORA standard (1000 g/hL pa) [16].

#### 3.2.6. Higher Alcohols

Samples were analyzed for six of the most common higher alcohols found in beverage alcohols: 1-propanol, 1-butanol, 2-butanol, isobutanol, isoamyl alcohol, and 1-hexanol. The concentrations of the six higher alcohols, summed together, ranged from 3.8–1200.1 g/hL pa, with the sum for one sample (#113) exceeding the AMPHORA upper limit of 1000 g/hL pa [16].

#### 3.2.7. Heavy Metals

Arsenic was detected in 29 of the samples (46.0%), cadmium in 47 of the samples (74.6%) and lead in 38 of the samples (60.3%). One sample (#125) contained arsenic concentrations of 113 μg/L, which was above the AMPHORA limit of 100 µg/L [16]. The concentrations of lead and cadmium were below the AMPHORA limits of 10 µg/L for cadmium and 200 µg/L for lead [16].

## 4. Discussion

Forty-nine of these 56 samples of unrecorded bai jiu from rural areas of China were within the AMPHORA guidelines for methanol, acetaldehyde, ethyl acetate, higher alcohols, and heavy metals. It is possible the samples may have contained other components which we did not test for that exceeded the guidelines.

The AMPHORA guidelines were chosen as the standards of comparison because they have been used in two previous studies of unrecorded alcohol in China [17,18] and because they provide guidelines for chemical standards not addressed in local or national regulations. For example, in China only methanol and one heavy metal (lead) are covered by regulations for bai jiu products [21,22].

Three of the 56 samples contained acetaldehyde amounts slightly above the AMPHORA guideline. In contrast, an earlier analysis of Chinese bai jiu samples found that 65.6% of samples exceeded the guideline [18]. The authors of that study suggested the high acetaldehyde levels may have been a result of unskilled distillers not discarding the first distillate. The earlier study’s samples came from a narrower geographic area. In light of these results from a wider geographic area, the disproportionately high number of high-acetaldehyde bai jiu samples found in the earlier study [18] may have been a localized phenomenon.

One sample of the 56 exceeded the AMPHORA guideline for higher alcohols. Higher alcohols are natural byproducts of fermentation; they are important to flavor and are not noted as significant health risks. Some studies have discussed higher alcohol being a potential health hazard in excessive amounts, and its effect is similar to that of ethanol [23,24,25,26]. However, Lachenmeier and Rehm in their editorial [26], stated that there is no evidence for the impact of higher alcohols in unrecorded alcohol on public health.

One sample exceeded the AMPHORA guideline for arsenic. The sample with the high arsenic result came from a site where the maker was installing new equipment; it is possible the arsenic was a contamination from the new construction. If the arsenic level continues to exceed guidelines it would impact the health of this maker’s regular customers over time.

Chinese regulations for bai jiu products made from grain limit methanol to not more than 0.6 g/L [21] and limit lead to not more than 0.5 mg/kg [22]. None of the samples in this study exceeded these limits. The absence of safety standards for beverage spirits should cause concern among public health officials.

Overall, the result of this analysis of Chinese unrecorded bai jiu samples indicates that consuming this type of unrecorded alcohol does not present health risks beyond the consequences of consuming ethanol. This is similar to the general conclusion made in the 2014 systematic review of studies of unrecorded alcohol from other countries around the world [4].

### 4.1. Ethanol

In China, drinkers prefer higher strength bai jiu (>40% ABV). Ethanol concentrations in 50/56 (92.6%) of the samples were in the range of 40–59% ethanol. The purposeful high-low ABV sampling may explain the presence of samples of ≥60% ethanol and may also explain why some of these samples had higher concentrations of ethanol than reported in the two previous studies of Chinese unrecorded bai jiu, which did not purposefully collect both high and low ABV samples [17,18]. The ABV for the recorded bai jiu samples purchased in Beijing ranged from 42.3–57.5%. In these samples there was no clear evidence that unrecorded bai jiu had a higher ABV than recorded alcohol.

### 4.2. Price, Labeling and Risk

The only systematic review of unrecorded alcohol studies [4] suggested that unrecorded alcohol is often cheaper than recorded alcohol, which enables a greater frequency and quantity of drinking, particularly among people with low incomes. The systematic review of studies [4] has also suggested that unrecorded alcohols have higher ABV than recorded alcohols. This study found that lower-priced unrecorded bai jiu was less potent than high-priced bai jiu. The least-expensive unrecorded bai jiu in this study was cheaper than the lower price range for recorded bai jiu. In rural areas of China, unrecorded bai jiu is easier to find than low-priced recorded bai jiu. The unrecorded bai jiu samples in this study had on average a higher ABV than the recorded bai jiu samples. The number of recorded bai jiu samples was small, however. Both of these characteristics (lower price and higher strength) have been mentioned by bai jiu drinkers as important reasons for preferring unrecorded bai jiu to recorded alcohol [12,27].

Another issue with unrecorded alcohol is whether the information that sellers give to customers about their product is honest or accurate. We found the majority of sellers accurately estimated the ABV of their product, despite not having the quality or strength confirmed by independent testing. Because high ABV bai jiu is preferred by customers and sells for a higher price, makers may have an incentive to over-state the ABV to customers. For the 20% of sellers who overstated the ABV by >2%, we can’t know if this was intentional or unintentional, but they were doing their customers little harm, other than potentially charging a higher price than their product warranted. Only eight sellers underestimated the ABV by more than 2%.

### 4.3. Placing Bai Jiu Consumption in a Cultural Context

Clearly the chemical composition of unrecorded bai jiu is the first consideration when assessing its potential risk to health; but the potential health consequences of alcohol consumption are strongly influenced by nonchemical factors, such as cultural and individual consumption patterns. These have not been widely explored in contemporary Chinese society. Historically, bai jiu is portrayed in Chinese visual and literary art works as important in social relationships, celebrations and worship of the gods. The reverence in Chinese culture for relationships, friendship, and connections is believed to be facilitated by bai jiu. Local makers of bai jiu accumulate loyal customers and consequently would not knowingly do anything to their products to undermine the reputation they have earned. So long as the supply chain from the maker to the drinker is short it is unlikely that contamination or adulteration would occur. Furthermore, Chinese drinking culture and attitudes have not been corrupted by colonialism. In other parts of the world, where people endured colonization by outsiders, alcohol was often used as a means of control, subjugation and reward, which established patterns of high-risk drinking that outlasted the colonial eras. In contrast, in China, the social controls, cultural norms and values related to alcohol are intact, and therefore Chinese drinking patterns are relatively low-risk compared to those of other regions of the world [1]. The WHO has suggested that policies to control unrecorded alcohol should accommodate cultural variations [7]. The intricate nature of alcohol culture in China, as well as the amount of local variation found in this large country means policy development and implementation should follow careful exploration of and understanding of the Chinese culture.

### 4.4. Limitations

The information available on the chemical composition of unrecorded alcohol in China is sparse. There is no claim that the results presented here are in any way representative of a country as large and as complex as China. The results of the comparison of unrecorded and recorded bai jiu price and ABV are especially tenuous as there were only seven recorded bai jiu samples. Nevertheless, these results add to the information on Chinese unrecorded bai jiu and suggest the significance of social and cultural aspects when discussing the production of unrecorded bai jiu. Together these initial results indicate the need for more sophisticated studies of larger representative samples in order to better describe the chemical composition of unrecorded bai jiu and to more accurately understand its production and use in the context of Chinese alcohol culture.

## 5. Conclusions

This study adds to our understanding of unrecorded bai jiu in China. It confirms findings of the two previous analyses of samples of unrecorded bai jiu in China, which have suggested that the components of the unrecorded bai jiu samples from China, with the possible exception of acetaldehyde, fell within guidelines for the components for unrecorded beverage alcohol adopted by the AMPHORA project. The main concern from Chinese unrecorded bai jiu is surrounding ethanol. The main concerns with ethanol consumption (whether from recorded or unrecorded) are alcohol dependency, alcohol poisoning, liver disease, certain types of cancer, traffic crashes, and family and social problems. Ethanol consumption is higher for drinkers who consume spirits, such as bai jiu, but is moderated by an individual’s and a culture’s values and drinking behavior patterns. The caution raised about acetaldehyde in one of the earlier studies was not confirmed in this study. However, the possibility of high levels of acetaldehyde in Chinese bai jiu deserves further attention for two reasons: Firstly that a significant proportion of Chinese drinkers prefers bai jiu to other types of alcohol beverages and secondly as much as half of the population may have an inherited trait that compromises acetaldehyde metabolism and increases the risk of certain cancers. This paper suggests the chemical composition and the social and cultural issues of unrecorded bai jiu consumption deserve consideration when making public policy.

## Supplementary Materials

The following are available online at http://www.mdpi.com/1660-4601/15/12/2710/s1, Table S1: Detailed table of the chemical test result for unrecorded and recorded Chinese bai jiu samples.

## Appendix A

**Chemical analysis**

Quantitative determination of volatile components was done by gas chromatography with a flame-ionization detector. Nitrogen was the carrier gas. Inductively coupled plasma optical emission spectrometry (ICP-OES) was used to determine the concentration of heavy metals (arsenic, cadmium, and lead). The concentration of ethanol was determined using an oenometer (alcoholometer). Volatile compounds contained in the samples are expressed in the unit of mg/L, whereas heavy metal components are reported in μg/L.

**Determination of Volatile Organic Compounds**

Gas chromatography was used to determine the concentrations of the following volatile organic compounds: Methanol, acetaldehyde, ethyl acetate, and higher alcohols. The test for each sample was recorded in mg/L. The apparatus consisted of a Shimadzu GC-2010, a PE Turbomatrix 16 (head space sampler), a flame ionization detector, and a OV1701 column. The operating conditions were: Injector temperature, 160 °C; detector temperature, 260 °C; column temperature, 40 °C (3 min)–180 °C (2 min)/10 °C; carrier gas, N2, 23 kPa; flow rate, 5 mL/min; air flow rate, 400 mL/min; H2 flow rate, 47 mL/min; make up gas, 30 mL/min; head space conditions, vial temperature: 70 °C; thermosetting time, 25 min; pressurization time, 25 min; transfer line temperature, 130 °C.

**Determination of Heavy Metals**

Samples were tested for three heavy metals: Cadmium (Cd), arsenic (As), and lead (Pb). Inductively coupled plasma optical emission spectrometry (Perkin Elmer Optima 8300 ICP-OES Spectrometer, PerkinElmer, Inc., Waltham, MA, USA) was used to determine the concentration of these metals, which are reported in units of μg/L. The standard reserve liquids of Cd, As and Pb (500 μg/L for each element) were purchased from national chemical reagent quality inspection center in China. The standard reserve liquids were diluted with 5% nitric acid (HNO_3_) to different concentrations for the establishment of standard curves.

For the purpose of quantitative analysis of heavy metals, spirit samples were digested first. For this, 19 mL of spirit sample was added with 1 mL nitric acid (HNO_3_) and then placed in the beaker, which was heated to remove alcohol and water from the sample. The heating was stopped when the volume of sample reached 1 mL. The ultrapure water was added to the digested sample to reach the 20 mL mark in the flask. Then, approximately 10 mL of the digested sample was placed in the reactor of the instrument to calculate the concentration of heavy metals.

**Determination of Alcohol by Volume Percent (ABV%)**

The concentration of ethanol is reported by the percentage alcohol by volume (ABV%). Alcohol strength was determined by an oenometer (alcoholometer). A 100 mL spirit sample was transferred into a 100 mL measuring cylinder. The oenometer was placed in the spirit sample and was allowed to float. The mark at which the spirit level crossed the stem of the instrument was recorded, and the concentration of alcohol or ethanol in sample was calculated according to temperature.

**Determination of pH**

A 10 mL spirit sample in a 50 mL clean beaker and a Sartorius PB-10 pH meter (Germany) was used for pH measurements.

**Caption for Graphical Abstract**

Unrecorded grain alcohol (酒) is ready to be ladled out of its traditional pottery storage jar into the customer’s container in a small market in China.
