# Supplementary material for: Chemical Composition and Safety of Unrecorded Grain Alcohol (Bai Jiu) Samples from Three Provinces in China
Source: Int J Environ Res Public Health. 2018 Dec 1;15(12):2710. doi: 10.3390/ijerph15122710 (PMC6313741; doi:10.3390/ijerph15122710)
Supplement: Supplementary file 1 [file ijerph-15-02710-s001.pdf]

Supplementary

TableS1. Detailed table of the chemical test result for unrecorded and recorded Chinese bai jiu samples.

**AMPHORA limit**						**1000 g/hL pa**	**50g/hL pa**	**1000 g/hL pa**	**1000 g/hL pa**	**200µg/L**	**100 µg/L**	**10 µg/L**
					g/hL pure alcohol (pa)[a]					µg/L		
type of alcohol	sample no.	cost (Yuan)	type of grain[b,c]	ethanol (ABV% from seller[c]	ethanol (ABV%)	methanol[d]	acetaldehyde[d]	ethyl acetate[d]	sum of higher alcohols[d]	lead[e]	arsenic[e]	cadmium[e]
昆明 Kunming	101	15	C	55	53.8	0.8	7.8	25.7	400.7	0.67	17.23	0.12
	102	30	5G	80	72.1	2.6	24.3	279.4	547.7	n.d.	12.79	0.28
	103	8	C	49	48	1.4	12.7	63.0	573.5	1.63	15.12	0.23
	104	15	B	55	53.4	7.2	66.6	59.0	466.2	10.00	n.d.	n.d.
	105	15	S	55	52.8	1.2	11.5	27.2	278.6	n.d.	n.d.	0.17
	106	12	5G	50	49.7	1.0	9.0	25.9	198.5	0.79	n.d.	n.d.
	107	6	S	50	50	1.1	9.8	23.3	254.2	10.20	n.d.	n.d.
	108	3.5	C	50	53.1	1.7	15.3	89.2	233.0	4.21	n.d.	1.56
	109	42	n/a	52	49.7	0.7	6.0	99.7	62.6	46.47	43.76	2.51
重庆 Chongqing	110	280	S	50	51.4	2.1	19.0	96.8	442.7	5.43	n.d.	n.d.
	111	35	S	60	54.5	3.0	27.7	133.0	406.0	15.80	22.39	n.d.
	112	60	S	50	50.4	2.3	21.5	94.5	430.4	n.d.	47.59	2.29
	113	80	5G	51	51	2.6	23.5	145.3	1220.1	14.58	n.d.	2.33
	114	4	5G	40	46.3	1.2	11.1	220.2	102.8	3.37	n.d.	0.51
	115	12	S	50	55.5	1.8	16.2	188.0	270.0	n.d.	n.d.	0.16
湖北咸宁 Hubei Xianning	116	5	R	51	50.4	5.8	53.7	87.7	175.8	25.36	55.42	0.08
	117	10	R	55	51	5.2	47.6	80.9	180.7	1.09	31.48	1.26
	118	5	R	49	48.6	6.5	60.0	64.8	106.4	13.46	n.d.	2.47
	119	15	B	50	49.4	1.5	13.5	133.3	133.4	n.d.	37.64	0.67

	120	30	S	52	52.4	1.8	16.3	57.5	85.5	8.11	n.d.	1.72
	121	8	S	48	47.3	2.0	18.3	24.7	76.3	n.d.	n.d.	1.73
	122	4	R	48	50.4	1.6	14.7	37.9	78.7	1.53	7.60	0.17
	123	20	B	51	52.2	1.5	13.4	73.8	262.7	3.78	n.d.	0.42
	124	10	S	n/a	49.4	3.0	28.1	103.9	30.7	n.d.	n.d.	0.44
	125	20	R	n/a	52.4	4.7	43.5	101.2	58.1	0.61	113.20	0.33
	126	8	R	51	51.4	1.3	12.4	19.8	81.4	20.35	n.d.	0.25
	127	20	R	50	51	0.9	8.3	43.4	116.2	13.23	n.d.	1.15
	128	8	R	51	50.7	1.5	13.4	40.1	215.9	9.95	n.d.	n.d.
	129	10	C	51	55.5	1.6	14.6	27.9	166.8	n.d.	n.d.	1.39
	130	10	R	48	54.5	1.5	13.7	56.5	540.3	13.88	n.d.	2.63
	131	10	S	50	51.8	2.3	20.7	129.6	109.2	n.d.	n.d.	1.25
	132	20	S	55	50.4	3.9	36.2	141.2	112.2	1.02	44.78	n.d.
	133	4	n/a	n/a	50.7	3.2	29.4	37.6	62.1	n.d.	24.88	0.31
	134	10	n/a	n/a	52.4	2.5	22.8	38.8	77.9	n.d.	52.42	1.06
	135	10	R	49	47	1.3	12.0	169.7	156.2	2.38	n.d.	2.25
	136	15	R	58	52.4	0.9	8.4	49.1	284.0	1.41	3.29	n.d.
	137	20	5G	60	55.1	1.6	14.8	113.7	92.9	n.d.	60.02	2.02
	138	20	R	60	54.1	3.7	34.4	311.8	569.4	n.d.	0.05	n.d.
	139	15	R	60	60.6	1.2	11.0	222.6	154.6	n.d.	22.71	0.98
安徽宣城 Anhui Xuancheng	140	20	S	46	48	0.5	4.4	86.8	441.9	n.d.	n.d.	0.99
	141	10	5G	45	46.3	0.5	4.6	77.0	413.0	n.d.	n.d.	n.d.
	142	10	n/a	50	50.7	0.3	2.9	91.9	904.3	9.27	n.d.	n.d.
	143	22	S	50	51.4	0.6	5.8	199.7	784.5	1.53	0.08	n.d.
	144	16	S	65	63.6	0.7	6.9	56.2	538.1	n.d.	1.37	0.89
	145	7	S	44	44.3	1.2	10.8	65.9	669.0	15.95	n.d.	1.08
	146	20	n/a	n/a	44.3	0.2	2.3	160.2	525.1	58.79	11.30	0.16
	147	7	S	40	40.8	1.3	12.4	138.3	727.2	n.d.	23.77	0.09
	148	15	S	47	53.7	0.8	7.5	366.5	168.7	6.48	n.d.	0.48
	149	20	5G	n/a	50.4	0.5	4.5	4.1	266.9	11.83	n.d.	0.59
	151	20	S	53	47.3	0.1	0.9	1.0	94.2	1.12	76.65	n.d.
	152	50	S	60	63.6	0.3	3.1	22.2	205.1	n.d.	n.d.	2.64
	153	60	R	60	53.4	0.2	1.4	61.1	588.5	1.68	n.d.	2.80

	154	60	RG	52	52.4	0.5	5.0	120.7	552.2	n.d.	37.95	1.98
	170	5	R	n/a	45.3	0.3	3.0	78.2	450.7	n.d.	33.62	0.93
北京 Beijing	155	14	S	65	60.6	0.6	5.9	118.1	458.7	0.32	n.d.	n.d.
	156	38	S	65	51.4	0.5	4.2	222.1	770.6	n.d.	4.99	0.20
	157[r]	8	n/a	50	47	0.9	8.6	143.6	632.4	18.46	94.85	0.43
	158[r]	6	n/a	56	56.8	0.5	4.3	230.6	3.8	21.46	n.d.	0.34
	159[r]	17	n/a	39	42.3	1.3	11.7	238.6	114.2	5.02	10.39	1.81
	160[r]	14	n/a	46	47.3	0.7	6.0	354.2	36.2	4.77	n.d.	0.19
	161[r]	6	n/a	53	54.5	0.6	5.4	265.8	41.2	n.d.	n.d.	0.69
	162[r]	13	n/a	56	57.5	1.3	12.0	204.0	164.9	n.d.	n.d.	n.d.
	163[r]	36	n/a	42	45.3	0.3	2.8	175.2	233.4	n.d.	3.02	n.d.

[a]Concentration of volatile compounds was obtained in mg/L and converted to g/hL pa by multiplying the obtained value by factor of 10/ABV for each sample

[b]Types of grain used in making alcohol: C- Corn; R-Rice; B-Barley; RG-Rice Gluton; S-Sorghum; 5G-Five grains

[c]n/a indicates not available. Information was not obtained from maker/seller or was not stated on label

[d]Detection limit: 10-3 mg/L for volatile compounds (methanol, acetaldehyde, ethyl acetate and higher alcohol)

[e]n.d. indicates not detected. Detection limit: 10-3 for non-volatile elements (Lead, Arsenic and Cadmium)

[r]recorded alcohol samples (n=7, sample#157 – #163)
